# The Weight of the Kidneys of the Horse

**Published:** 1902-05

**Authors:** J. T. Duncan

**Affiliations:** Professor of Anatomy, Ontario Veterinary College, Toronto, Canada


### THE WEIGHT OF THE KIDNEYS OF THE HORSE.

BY J. T. DUNCAN, M.D., V.S.,
PROFESSOR OF ANATOMY, ONTARIO VETERINARY COLLEGE, TORONTO, CANADA.

FOR a number of years we have made it a rule at the Ontario Veterinary College to keep a record of the principal points of interest in regard to each subject dissected. Among the points noted are the weight of certain organs. These are weighed by the students who are on the subject, entered by the secretary of the class on forms provided for the purpose, verified by the demonstrator of anatomy, and returned to the college after the session has closed.

A study of these records often brings out interesting points. For instance, in looking over the columns showing the weight of the kidneys, it was noticed—

1. That these organs vary in weight more than is usually stated.
2. That this variation may be strikingly shown in the right and left kidney of the same animal.
3. That the left is not infrequently heavier than the right.
4. That a given series of cases may show that the left approximates closely to the right in weight.

The number of horses dissected during the past session (excluding cases of disease) was 54. Out of these 54 cases tabulated, the heaviest kidney was found to have weighed forty ounces, the lightest but sixteen ounces, thus showing very considerable variation—for it would take two and one-half of the smaller organ to make the larger.

In reference to the second point—the differing weight of the right and left kidney—the list shows it well. Perhaps the most striking case is one in which the right weighed forty ounces, the left but twenty-five ounces.

The third point is illustrated by the fact that in one case the left kidney weighed thirty-eight ounces, the right but twenty-five ounces; or, a difference of thirteen ounces in favor of the left.

It is, however, when we come to the average weights that the most interesting point is brought out. Chauveau, in his work on *Comparative Anatomy*, says "the right kidney (of the horse) is always more voluminous and heavy than the left, its average weight being twenty-seven ounces, while that of the left is twenty-five ounces." Other authorities, while not finding the organs so heavy as does Chauveau (Bradley's weights are: right kidney, twenty-one ounces; left, nineteen ounces), agree that the right is about two ounces heavier than the left. In a series of cases published some years ago, our results were (from memory): right, twenty-three and a half ounces; left, twenty-one and a half ounces. All the more remarkable, these are the results from the present series, the average weight being for the right, $23\frac{29}{54}$ ounces; for the left, $23\frac{17}{54}$ ounces; or, approximately, right, $23\frac{1}{2}$ ounces; left, $23\frac{1}{3}$ ounces.

These results are surprising, but are obtained from—comparatively—so small a number of cases that they but point to the need for further examination.

In man, the right and left kidneys each average four and a half ounces.

